# Patient engagement for the development of equity-focused health technology assessment (HTA) recommendations: a case study of two Canadian HTA organizations

**DOI:** 10.1017/S0266462325000182

**Published:** 2025-03-28

**Authors:** Rosiane Simeon, Abdulah Al Ameer, Shehzad Ali, Kumanan Wilson, Janet H. Roberts, Ian D. Graham, Peter Tugwell, Vivian A. Welch

**Affiliations:** 1 Interdisciplinary School of Health Sciences, University of Ottawa, Ontario, Canada; 2 Bruyère Research Institute, Bruyère Continuing Care and University of Ottawa, Ottawa, Ontario, Canada; 3 Department of Epidemiology and Biostatistics, Western University, Ontario, Canada; 4 Department of Medicine, University of Ottawa, Ontario, Canada; 5 School of Epidemiology and Public Health, University of Ottawa, Ontario, Canada

**Keywords:** health equity, Health Technology Assessment (HTA) equity-oriented healthcare, patient engagement, HTA decision-making, equity-focused HTA recommendations, Canadian HTA

## Abstract

**Background:**

Health technology assessment (HTA) is a form of policy analysis that informs decisions about funding and scaling up health technologies to improve health outcomes. An equity-focused HTA recommendation explicitly addresses the impact of health technologies on individuals disadvantaged in society because of specific health needs or social conditions. However, more evidence is needed on the relationships between patient engagement processes and the development of equity-focused HTA recommendations.

**Objectives:**

The objective of this study is to assess relationships between patient engagement processes and the development of equity-focused HTA recommendations.

**Methods:**

We analyzed sixty HTA reports published between 2013 and 2021 from two Canadian organizations: Canada’s Drug Agency and Ontario Health.

**Results:**

Quantitative analysis of the HTA reports showed that direct patient engagement (odds ratio (OR): 3.85; 95 percent confidence interval (CI): 2.40–6.20) and consensus in decision-making (OR: 2.27; 95 percent CI: 1.35–3.84) were more likely to be associated with the development of equity-focused HTA recommendations than indirect patient engagement (OR: .26; 95 percent CI: .16–.41) and voting (OR: .44; 95 percent CI: .26–.73).

**Conclusion:**

The results can inform the development of patient engagement strategies in HTA. These findings have implications for practice, research, and policy. They provide valuable insights into HTA.

## Background

Health equity involves the fair distribution of health outcomes across all population groups (1;2). Decision-makers can achieve health equity by improving health outcomes through addressing social determinants of health, such as access to resources and discrimination within and outside the healthcare system (1;2). Researchers suggest various tools to support health equity, including knowledge production (3), practice guidelines (4), and policy analysis (5). Health technology assessment (HTA) is a form of policy analysis that informs decisions about funding and scaling up health technologies (6;7). Health technologies are inherent in health service infrastructure and include diagnostic, preventive, treatment, and rehabilitation procedures to support health and well-being (6;7). Organizations such as Canada’s Drug Agency (CDA) and Ontario Health develop HTA recommendations by reviewing evidence on health technologies to ensure their safety, effectiveness, and compliance with broader ethical, social, and legal standards (6;7).

Equity-focused HTA recommendations explicitly address the impact of health technologies on individuals disadvantaged in society due to specific health needs and social determinants, such as those in the PROGRESS-Plus framework (5;8). PROGRESS-Plus stands for place of residence, race/ethnicity, occupation, gender, religion, education, socioeconomic status, social capital, and reported strata, such as sexual orientation and individuals with disabilities (8). It was developed to facilitate identifying and integrating health equity factors in interventions, research, and policy (8).

Patient engagement involves collecting input to influence knowledge creation, such as HTA recommendations (9;10). HTA organizations can collect patient input through direct engagement, where analysts engage individual patients, or indirect engagement, where patient organizations compile member input for submission to HTA agencies (9;11). Both types of engagement aim to ensure that HTA recommendations reflect patient experiences (9;11). Patient engagement is increasingly recognized as essential in HTA processes to incorporate diverse perspectives, particularly from underrepresented and disadvantaged groups (12–14). By involving patients in their HTA process, HTA organizations can better understand the needs, preferences, and experiences of those most affected by health technologies (9;13).

**Figure 1.**
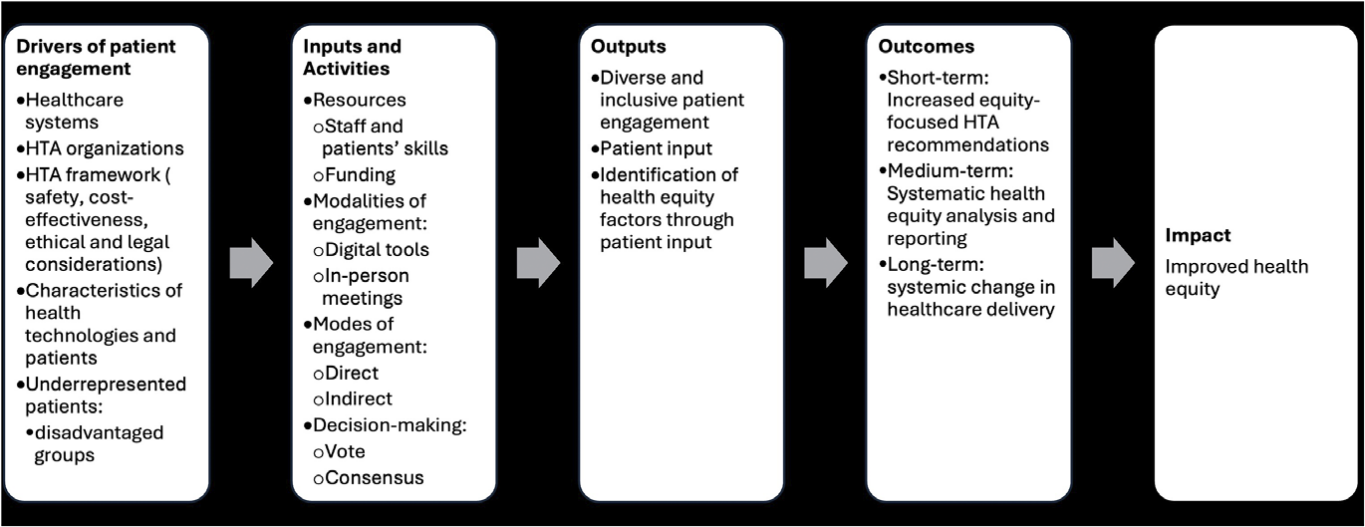
Logic model describing how patient engagement influences the development of equity-focused HTA recommendations.

The logic model in Figure 1 outlines the theory of change, demonstrating how patient engagement may influence the integration of equity considerations into HTA recommendations. It identifies key drivers of patient engagement, including healthcare systems, HTA organizations, HTA frameworks, and the characteristics of health technologies and patient populations. Human and financial resources, such as skilled staff, funding, and diverse engagement modalities – including digital tools and in-person meetings – can facilitate direct and indirect patient engagement. Decision-making models, such as consensus and voting, can assist in identifying and incorporating health equity factors through patient input. Patient engagement outcomes may vary from increases in equity-focused HTA recommendations to systemic changes in healthcare delivery, ultimately contributing to improved health equity. A complete description of the logic model can be found in Supplement 1.

It is worth noting that patient engagement is just one approach to developing equity-focused HTA recommendations (15;16). The significance of patient engagement and equity considerations in recommendations varies with HTA practices, which are impacted by local governance structures, healthcare priorities, and population needs (17–19). Panteli et al. (20) highlighted the variability in addressing health equity in HTA practices, pointing to a need for standardized approaches and methodological guides to enhance the integration of health equity factors in HTA.

In addition, recent studies have revealed the need to improve inclusivity in patient engagement to enhance their impact on health equity (12;13). There is limited evidence on which patient engagement processes best support the incorporation of health equity factors in HTA (9;13;21). Additional research can help identify best practices to strengthen patient engagement’s impact on advancing health equity and improve patient engagement structures to guide equity-focused HTA recommendations (22–24). Decision-makers use HTA recommendations to inform policies such as drug coverage, healthcare services, preventive interventions, and public health workforce training, all of which have equity implications when rolled out to the public.

## Objectives

The study aims to bridge existing research gaps by examining the association between patient engagement processes and the development of equity-focused HTA recommendations. By clarifying these relationships, the study will provide insights into best practices for integrating patient concerns in HTA recommendations, ultimately contributing to more equitable healthcare outcomes (12;13;21;25). In this article, we addressed the following research questions:

- What are the characteristics of equity-focused HTA recommendations?
- What patient engagement processes are associated with equity-focused HTA recommendations?

## Methods

### Study design

We used a cross-sectional case study design to assess the prevalence of equity-focused HTA recommendations and to determine the relationships between patient engagement processes and equity-focused HTA recommendations using a sample of 60 reports from two Canadian HTA organizations. Case studies help generate an in-depth understanding of a complex issue in its natural setting (26;27). The case here consists of patient engagement processes in two Canadian organizations, CDA and Ontario Health, operating at the provincial and federal levels. We decided to use an explanatory case study approach because it can help generate theories about the influence of patient engagement processes on incorporating equity factors in recommendations based on the context of HTA (28).

For example, the HTA process in Ontario is influenced by the provincial government’s emphasis on addressing local healthcare challenges, such as access to services in rural and remote areas (29). This focus may lead Ontario Health to prioritize patient engagement methods that capture the voices of those who might be underrepresented in health research, such as rural populations and patients with rare conditions. Meanwhile, CDA’s broader national mandate means that HTA processes might only sometimes capture such localized nuances (29).

### Sample size calculation

We used a purposeful sample of sixty HTA reports from CDA and Ontario Health. We decided on Canadian HTA because research shows that HTA practice is context-bound, with patient engagement for health equity analysis varying significantly across organizations and countries (11;17–20). This context specificity implies that including reports from noncomparable settings may compromise the accuracy of the findings and restrict their generalizability (30). For example, in HTA organizations where people discuss democratic rights, the focus may be on implementing patient engagement that considers diverse representation and meaningful participation to clarify choices, usage, and fair distribution of health technologies (15;31;32). In other political systems, HTA organizations may concentrate their patient engagement on building consensus around using and covering health technologies (19;33;34). HTA practices in CDA and Ontario Health are based on the same Canadian democratic political system (17;31;35). This example emphasizes the need to understand the context of HTA practices to ensure the study’s recommendations are relevant and actionable.

We calculated the sample size based on adequacy for logistic regression, drawing on existing literature and prior studies (30). We used an earlier study that analyzed equity factors in nineteen HTA agencies (36). The study found that around 50 percent of the HTA agencies considered equity factors through their methods or analysis of legal and ethical issues (36). Also, another study that examined equity considerations in the World Health Organization (WHO) guidelines showed that only 25 percent of the guidelines contained PROGRESS-Plus items (37). We expected HTA to include more equity factors than the WHO guidelines because HTA must consider the local context in its analysis of health technologies. In contrast, WHO guidelines require further adjustment before their implementation in a country. So, we used a 40 percent ratio, giving a sample size of fifty. We increased the sample size to sixty reports to account for variability and ensure robustness.

### Identification of eligible reports

HTA reports had to meet three main criteria to be included in this study. First, HTA organizations must involve patients in creating the reports. Second, the reports should have clear recommendations, but they were not required to contain health equity factors in their recommendations. Third, eligible HTA reports must have been published between 2013 and 2021. Reports were excluded if healthcare providers provided input on behalf of the patients, if patient experience reviews were used as a substitute for patient input, or if reports did not include any patient input. RS identified the HTA organizations and the HTA reports. RS and AA screened all the reports for eligibility. Table 1 provides a summary of the included reports.

**Table 1.** Characteristics of included reports

Characteristics	Description	*n* (%)
Year of publications		
2013–15	Earlier implementation period	9 (15%)
2016–21	Recent implementation period	51 (85%)
Types of HTA review		
pan-Canadian Oncology Drug Review (pCODR)	CDA reports focused on cancer drugs	15 (25%)
Common Drug Review (CDR)	CDA reports focused on noncancer drugs	25 (42%)
Ontario Health	Ontario Health reports focused on medical devices and virtually delivered health technologies	20 (33%)

Using stratified sampling, sixty reports were randomly selected across the three categories: twenty-five from the Common Drug Review (CDR), fifteen from the pan-Canadian Oncology Drug Review (pCODR), and twenty from Ontario Health. We selected reports based on types of HTA reviews, years of publications, and patient engagement. Contrary to Ontario Health, which did not categorize HTA products on its website, CDA had several HTA products. Two CDA products were selected: the CDR and the pCODR. The term “common drugs” designates health technologies in the CDA CDRs focused on conditions such as hypertension, diabetes, and asthma. For Ontario Health, we considered HTA reports that cover medical devices and virtually delivered health technologies.

We considered the abovementioned reports because of their potential for health equity implications. For instance, certain common drugs cover health conditions such as diabetes and hypertension, which disproportionately affect some population groups in Canada (38). Oncology drugs may require more frequent interactions with health systems for monitoring than some nononcology drugs (21). Sufficient scientific evidence may not exist on technologies targeting rare diseases, making the patient experience a critical source of evidence in formulating recommendations for these conditions (39). Virtually delivered health technologies may not be accessible to those with limited access to digital technologies (40). Medical devices may raise concerns about access and adjustment to individual needs (41).

We selected the 2013–21 period to identify HTA reports before establishing the Patient and Community Liaison Forum in 2013. This forum was created to improve patient involvement in HTA processes in CDA (31). Ontario Health began including patient input in its HTA reports in 2015.

Using stratified sampling enhances the sample’s representativeness by including all relevant HTA reports (42). This reduces selection bias, increases the validity and reliability of the findings, and improves their generalizability to broader HTA practices within Canada and internationally (42). In Supplement 2, we describe the process of selecting the reports.

We did not consider HTA reports on digital health technologies. Digital health technologies are different from digital technologies, which we assessed as a modality of patient engagement. Digital health technologies encompass medical devices with built-in digital systems that support various functions in healthcare, including drug administration, diagnostics, monitoring, and predictive testing (43). We excluded them because there is limited patient engagement in HTA regarding those health technologies (43).

### Screening and data extraction

During the screening phase, reports were carefully reviewed to confirm the presence of patient engagement and HTA recommendations. Three reports were excluded due to the absence of patient engagement: one included feedback from healthcare providers only, one was based on a literature review of patient experiences, and one did not contain patient input at all. The three reports were replaced to maintain the sample’s integrity: two from CDR and one from Ontario Health. Studies were not screened based on the presence of health equity factors in their recommendations. The final sample included sixty HTA reports that met the study’s criteria.

We developed a data extraction form using items from the PROGRESS-Plus framework (8), the checklist to guide equity considerations in HTA (5), and the published literature on characterizing health equity factors in studies (44;45). We described patient engagement activities using items from the practical guidance for involving stakeholders in health research (46). A single reviewer (AA) extracted data in the included HTA reports and the first author (RS) checked the extracted data for quality control. We provided detailed descriptions of the variables of interest in Supplement 2.

### Data management and analysis

We used Microsoft Excel for descriptive analysis and the R software package for inferential analysis (47). We utilized Pearson’s *χ* ^2^-test to determine the degree of associations between patient engagement processes and equity-focused HTA recommendations (95 percent confidence interval (CI), *p* = .05). We used logistic regression to examine the direction and strength of associations between patient engagement processes and equity-focused HTA recommendations. These are dichotomous variables, which take the value of 1 when the criteria are present and zero otherwise. We expected the coefficient for direct patient engagement or the consensus decision-making model to be >0 and statistically significant. Therefore, we will reject the null hypothesis if neither the types of patient engagement nor the decision-making models have a relationship with the likelihood of equity-focused HTA recommendations.

We performed a regression analysis to determine the association between patient engagement processes and equity-focused HTA recommendations. We did not add a variable for the three different types of reviews. We did not expect the implementation of patient engagement to differ across the two organizations. For example, if Ontario Health or CDA implemented direct engagement, they would do it similarly. We then calculated the odds ratio (OR) to determine the likelihood of identifying equity-focused recommendations for each type of patient engagement and decision-making model.

## Results

### Overview of patient engagement processes

*Types of patient engagement.* The analysis of sixty HTA reports from CDA and Ontario Health revealed diverse patient engagement processes, highlighting direct and indirect methods. Ontario Health mainly used direct engagement. Indirect engagement, primarily used by CDA, involved receiving patient input through submissions from patient organizations. Indirect engagement accounted for 67 percent of the sample. Some reports (12 percent) included patient and healthcare provider input.

*Modes and modalities of engagement.* The modes of engagement varied between interviews, surveys, and mixed methods. All the patient input in the Ontario Health reports was collected through interviews. In contrast, among the patient organizations submitting input to CDA, 55 percent reported their methods of gathering feedback. Digital technologies were the primary modality for engaging patients. Ontario Health and CDA employed digital tools, such as online surveys, discussion boards, and social media, to facilitate engagement.

*Decision-making models and patients’ roles.* The decision-making models identified in the reports included consensus meetings and voting. Consensus was the predominant decision-making model used in 58 percent of the HTA processes, particularly within Ontario Health and the pCODR. Voting was utilized primarily in the CDR processes, accounting for 42 percent. Patients contributed as key informants or members of advisory committees and participated in decision-making sessions. Supplement 3 provides additional information on the characteristics of patient engagement processes.

### Identification of equity-focused HTA recommendations

We defined an equity-focused HTA recommendation as containing at least one PROGRESS-Plus item. Some Ontario Health reports explicitly referred to health equity, but the CDA reports did not have a section on health equity. For HTA recommendations, we recorded PROGRESS-Plus items in the rationale and the evidence used to inform the HTA recommendations. This allowed us to categorize a maximum number of HTA reports containing health equity factors. Our approach to identifying equity-focused recommendations in the HTA reports ensures that we remain inclusive in our coding.

For example, if PROGRESS-Plus items were recorded in the HTA recommendations only, less than a third (28 percent) of the included HTA reports would be classified as containing health equity factors compared to 68 percent when using the abovementioned procedures. When a PROGRESS-plus item was repeated more than once in either section, we counted this item as one mention to avoid overrepresentation. We identified PROGRESS-Plus items in the reports’ patient input (55 percent) and HTA recommendations sections (68 percent). HTA and patient organizations have not provided details on how they incorporated equity considerations into patient input and recommendations.

We identified twelve unique PROGRESS-Plus items across all the included HTA reports, six of which were from the PROGRESS category. These consisted of a place of residence, language, gender, education, socioeconomic status, and social capital. We coded the other six items in the “Plus” category. They consisted of affordability, age, ethical issues, severity of conditions, treatment logistics, and stigma. We recorded stigma, social capital, and gender in patient input only. We did not find the following items from the PROGRESS framework in any sections of the included HTA reports: race/ethnicity/culture and religion.

### Health equity factors in patient input and HTA recommendations

**Figure 2.**
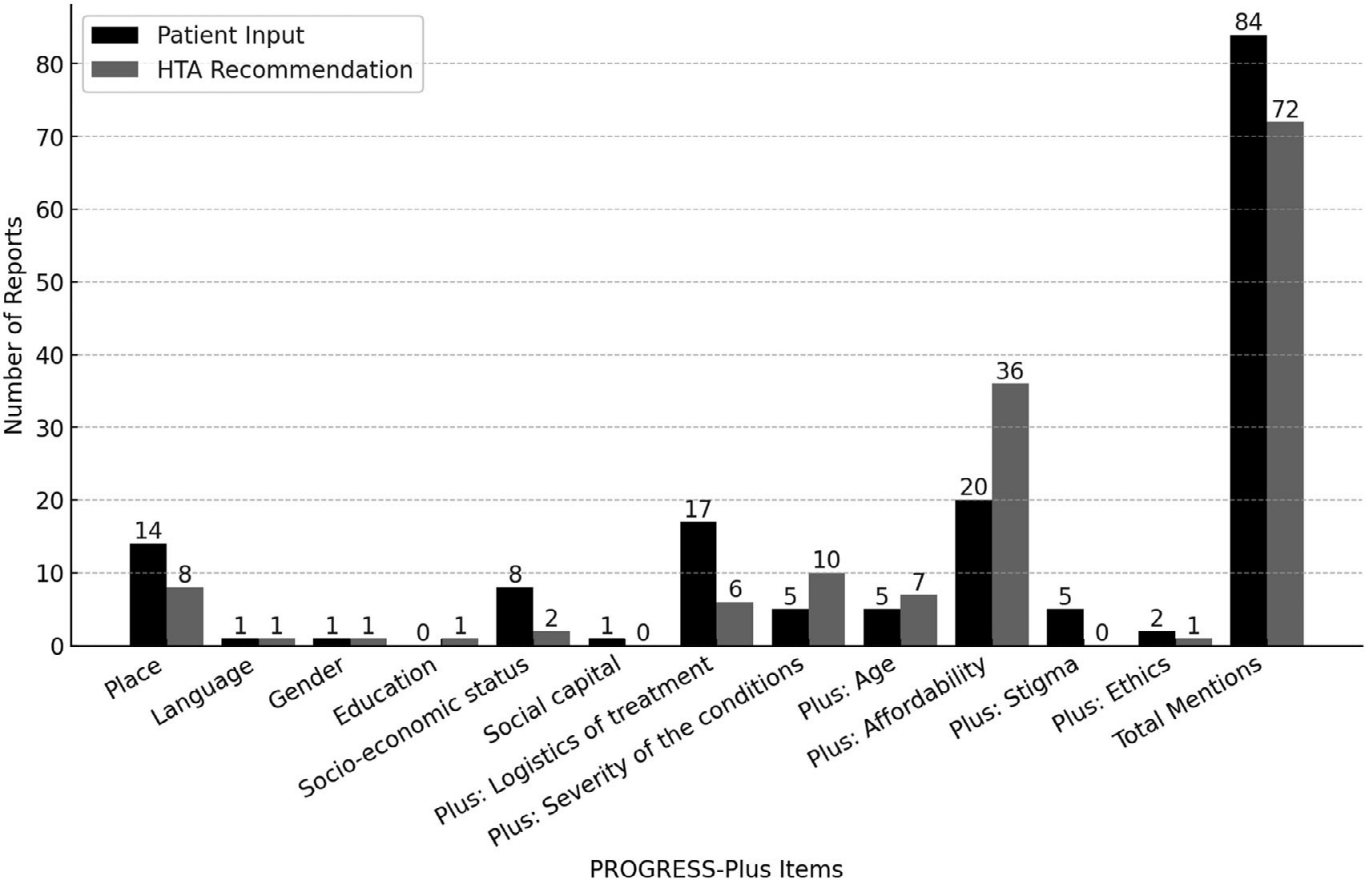
Mentions of PROGRESS-Plus items in the included reports.

We compared the number of PROGRESS-Plus items identified in patient input with those recorded in HTA recommendations. Figure 2 displays the PROGRESS-Plus items found in the included reports. As shown in Figure 2, mentions of PROGRESS-Plus items were more common in the patient input section (eighty-four mentions) than in the HTA recommendation section of the reports (seventy-two mentions). We identified eight PROGRESS-Plus items common to the reports’ patient input and HTA recommendation sections. However, there were differences in how these factors were represented in patient input compared to HTA recommendations. For example, affordability was the most frequently cited factor in patient input and recommendations, appearing in 60 percent (twenty out of thirty-three) of the patient input but increasing to 37 percent (thirty-six out of forty-one) in the HTA recommendations. Conversely, treatment logistics were highlighted in 51 percent (seventeen out of thirty-three) of the patient inputs but dropped significantly to 15 percent (six out of forty-one) in the recommendations.

### Association between patient engagement and equity-focused HTA recommendations

We used the R package for statistical analysis (47). As shown in Table 2, we found that HTA reviews that used direct patient engagement (OR: 3.85; *p*-value = .0007; 95 percent CI (2.40–6.20)) and consensus for decision-making (OR: 2.27; *p*-value = .002; 95 percent CI (1.35–3.84)) were more likely to result in equity-focused HTA recommendations. On the other hand, the likelihood of developing equity-focused HTA recommendations was lower with indirect patient engagement (OR: .26; 95 percent: .16–.41) and voting in decision-making (OR: .44; 95 percent: .26–.73), respectively.

**Table 2.** Inferential statistics

Dependent variable: equity-focused HTA recommendation	Regression coefficients	Odds ratio (OR)	Standard error	*p*-values	95% CI of odds ratio
Types of patient engagement					
Direct engagement	1.35	3.85	.23	.0007	2.40–6.20
Indirect engagement	−1.35	.26	.23	.0007	.16–.41
Models of decision-making					
Consensus	.82	2.27	.26	.002	1.35–3.84
Voting	−.82	.44	.26	.002	.26–.73

More specifically, the likelihood of recording equity-focused HTA recommendations was 2.27 higher when HTA advisory committees used consensus to make HTA decisions than when they used to vote. This scenario was noted in Ontario Health and pCDOR, with the difference that patient organizations submitted the patient input for pCODR reviews. The likelihood of recording equity-focused HTA recommendations in Ontario Health was generally 3.85 higher than the other HTA reports.

## Discussion

We examined sixty reports from two HTA Canadian organizations to study the association between patient engagement and incorporating equity factors in HTA recommendations. HTA organizations used direct and indirect engagement to collect patient input to inform effectiveness analysis and recommendations. Patients and HTA organizations engaged patients through digital and in-person modalities. However, patient organizations used a more comprehensive range of methods to engage patients than HTA organizations. Patients contributed to developing recommendations by participating in consensus and voting as members of HTA advisory committees.

We used a broad definition to help capture health equity considerations in the HTA reports. The results suggested that patient engagement played a role in incorporating health equity factors in the included reports. The findings also showed that combining specific patient engagement procedures might increase the identification of health equity factors to inform HTA recommendations. As in previous studies, the results indicated that direct engagement and consensus in decision-making increase the integration of health equity factors in HTA (14;48). For example, HTA advisory committees that used consensus as their decision-making model were more likely to consider equity factors in their recommendations. Ontario Health and pCODR used consensus as their decision-making model. However, HTA analysts directly interviewed patients to collect input for Ontario Health, whereas patient organizations submitted input for pCODR reviews.

The findings also align with previous research, which suggested that the context of HTA practices may influence health equity reports in HTA recommendations (18;20;29). Health equity factors in the pCODR reviews, which used consensus for decision-making, could be linked to the history of sustained advocacy around oncologic treatments (21). Similarly, a lack of awareness and organized advocacy around certain conditions in the CDR pool could explain why PROGRESS-Plus items were less likely to be mentioned in those reports. CDR covers conditions, such as diabetes, hypertension, mental health, and some rare diseases, that are known to disproportionately affect racialized individuals, women, historically stigmatized conditions, and people underrepresented in research (38).

The reviewed HTA recommendations did not identify critical factors such as gender, sex, occupation, race/ethnicity, and religion. This oversight may limit the potential of HTA recommendations to address health equity. A comprehensive health equity analysis must account for the compounded disadvantages that patients experience at the intersection of multiple marginalized identities (13;49;50). Earlier studies showed that gender, culture, access to social capital, and discrimination significantly impact health inequities (2;38). This emphasizes the need to discuss the various and interconnected challenges affecting the distribution of resources and health outcomes across population groups (2;49;50). Integrating frameworks, such as PROGRESS-Plus (8), intersectionality (50), and structural violence (49), can strengthen health equity analysis in HTA. This integration ensures that HTA analysts consider patients’ diverse needs and systemic barriers to inform HTA recommendations, effectively promoting health equity (2;29;50).

### Strength and limitations

The study addresses several gaps identified in previous research concerning the characteristics of patient engagement and health equity considerations within HTA practices in Canada and abroad (12;13;20;29). It spotlights patient engagement as an intervention with distinct processes that might influence incorporating equity factors in HTA recommendations. Earlier studies have highlighted the need for standardized approaches to developing equity-focused HTA recommendations (20;23). Using established frameworks like PROGRESS-Plus to identify equity factors in HTA recommendations offers a replicable method for other HTA organizations to improve their focus on health equity. The study helps demonstrate the application of the conceptual framework to identify health equity factors in HTA recommendations.

Despite these strengths, many limitations are worth considering before utilizing the research findings. The sample size might lead to missing HTA reports with more health equity considerations. We only conducted the study with two agencies in Canada. We cannot know if it applies to other agencies as their contexts differ. However, our hypothesis can be tested in other HTA settings. We did not add a variable for the three types of HTA review to help increase the power of the analysis. When conducting this research, we could not find a taxonomy of health technologies. As a result, we did not categorize the types of health technologies into pharmaceutical and nonpharmaceutical. If there were a difference due to the types of HTA reviews and health technologies, we would not be able to assess it. Furthermore, the data were extracted by a single reviewer, and variables were not independent in the analysis. To help reduce errors in data extraction, the first author checked for quality control. Finally, we cannot know how much advisory committee members weigh health equity factors in their final decision.

### Implications for practice, policy, and research

HTA and patient organizations can utilize these findings to improve patient engagement and promote health equity analysis. The findings can help develop patient engagement strategies and raise public awareness about the importance of patient input in HTA. Patient advocates can use these results to support their efforts in advocating for increased inclusion of their perspectives in HTA recommendations and collaborate with HTA organizations on patient input reporting structures. The findings have implications for policy-makers who can use them to initiate discussion about expectations of health equity factors in HTA recommendations for their jurisdictions. Future research could investigate the impact of equity-focused HTA recommendations on health systems, including funding decisions regarding health technologies. Other studies may explore the implications of applying a health equity lens to the HTA process, from scoping to developing recommendations, including using tools to move from evidence to decision-making.

### Conclusion

This study is the first to explore how patient engagement processes influence the development of equity-focused HTA recommendations in CDA and Ontario Health. The findings suggest that direct patient engagement with HTA analysts leads to a greater focus on equity considerations in recommendations. The study highlights the need for closer collaboration between HTA organizations and patients to ensure that patient perspectives are included. This research sets the stage for further exploring approaches to developing equity-focused HTA recommendations in partnership with patients. It offers insights for HTA and patient organizations to educate the public on contributing to healthcare system design for enhancing health equity.

## Supporting information

Simeon et al. supplementary material Simeon et al. supplementary material

## Supplementary material

The supplementary material for this article can be found at http://doi.org/10.1017/S0266462325000182.
