# Supplementary material for: Patient engagement for the development of equity-focused health technology assessment (HTA) recommendations: a case study of two Canadian HTA organizations
Source: Int J Technol Assess Health Care. 2025 Mar 28;41(1):e27. doi: 10.1017/S0266462325000182 (PMC12086597; doi:10.1017/S0266462325000182)
Supplement: Simeon et al. supplementary material [file S0266462325000182sup001.zip › Supplement 1_Description of Logic Model_PE and Equity-focused HTA Recommendations_Feb.19.docx]

**Theory of Change: Description of Logic Model**

This logic model in Figure 1 underscores the importance of integrating patient engagement into health technology assessment (HTA) processes to identify and address health equity factors. By systematically involving patients, particularly those from underrepresented groups, HTA recommendations can be better tailored to address the population's diverse needs, ultimately driving more equitable healthcare decisions (1,2). It is important to note that health equity can be integrated through multiple strategies, including legal, ethical, and social determinants, patient engagement and cost-effectiveness analysis (3,4). This logic model is action-oriented and not agent-based; it does not seek to explain causal relationships and variations between components.

Figure 1: A Logic Model Illustrating How Patient Engagement Can Influence Equity-Focused HTA Recommendations.

**Drivers of Patient Engagement for Health Equity in HTA:**

- **Healthcare Systems:** The broader healthcare environment, which includes the public and private sectors, can influence patient engagement processes. Systems' emphasis on equity and patient-centred care drives HTA organizations to include patient voices in their decision-making processes (6–8).
- **HTA Organizations:** Organizations are critical in setting patient engagement standards. They guide how patient input is incorporated into HTA processes, impacting the health equity analysis and the development of equity-focused HTA recommendations.
- **HTA Framework:** The frameworks used by HTA organizations impact the structure and approach for integrating equity considerations into technology assessments (9).
- **Characteristics of Health Technologies and Patients:** Health technologies' specific features, such as accessibility and usability, affect their relevance to different patient groups. Similarly, patients' socio-demographic characteristics influence their engagement level and input relevance.
- **Underrepresented and Disadvantaged Patients:** Ensuring the inclusion of underrepresented and disadvantaged groups in HTA processes can help develop recommendations that address diverse health needs (10,11).

**Inputs and Activities:**

- **Resources:**
  - **Staff and Patients’ Skills:** The skills and competencies of both HTA staff and patients are crucial for effective engagement. Training in cultural competencies and digital literacy can enhance the engagement process (12,13).
- **Funding:** Adequate funding supports diverse engagement activities, compensates participants, and ensures the sustainability of patient engagement initiatives (12,13).
- **Types of Engagement**: This defines how HTA organizations collect patient input.
- Direct Engagement: Involves HTA organizations in collecting patient input from individual patients through interviews, surveys, or focus groups (10,13,14).
- Indirect Engagement: This involves HTA collecting patient input through patient organizations, which conduct various engagement activities (e.g., interviews, surveys, and focus groups) with their members to gather feedback to submit to HTA organizations (10,13,14).
- **Modalities of Engagement:** Refers to the means used to facilitate engagement, such as digital platforms, telephones, or in-person meetings. Different modalities of engagement can affect patients’ participation and the quality of feedback provided, which may influence the incorporation of patients’ concerns in HTA recommendations (11,15).
- **Decision-Making Models:** This section outlines how HTA organizations integrate patient input into HTA recommendations. The decision-making approaches illustrate the extent to which patients influence the shaping of HTA recommendations. Generally, consensus offers more opportunities for impacting decision-making than voting(11,15).

**Outputs:**

- **Diverse and Inclusive Patient Engagement:** Engaging a broad range of patients ensures that HTA processes are inclusive and consider various health equity factors (16–18).
- **Patient input:** Patient input provides valuable insights into the lived experiences of those affected by health technologies, which informs equity considerations in HTA (16–18).
- **Health Equity Factors through Patient Input:** Systematic patient engagement helps identify equity-related issues, such as access barriers and differential health outcomes anchored in social determinants of health (16–18).

**Outcomes and Impact:**

- **Short-Term:** Increased focus on health equity in HTA, resulting in more recommendations that explicitly address the needs of disadvantaged populations (16–18).
- **Medium-Term:** Establishing systematic health equity analysis and reporting practices within HTA organizations, leading to more consistent inclusion of health equity considerations (16–18).
- **Long-Term Outcomes and Impact:** Achieving systemic change in healthcare delivery that prioritizes health equity, ultimately leading to reduced health disparities and more equitable health outcomes for all (16–18).

**References**

1. Benkhalti M, Espinoza M, Cookson R, Welch V, Tugwell P, Dagenais P. Development of a checklist to guide equity considerations in health technology assessment. I*nt J Technol Assess Health Care*. 2021;37(gti, 8508113):e17.

2. O’Neill J, Tabish H, Welch V, Petticrew M, Pottie K, Clarke M, et al. Applying an equity lens to interventions: Using PROGRESS ensures consideration of socially stratifying factors to illuminate inequities in health. *J Clin Epidemiol* [Internet]. 2014;67(1):56–64. Available from: http://dx.doi.org/10.1016/j.jclinepi.2013.08.005

3. Cookson R, Mirelman AJ, Griffin S, Asaria M, Dawkins B, Norheim OF, et al. Using Cost-Effectiveness Analysis to Address Health Equity Concerns. *Value Health J Int Soc Pharmacoeconomics Outcomes Res*. 2017 Feb;20(2):206–12.

4. Snow ME, Tweedie K, Pederson A. Heard and valued: the development of a model to meaningfully engage marginalized populations in health services planning. *BMC Health Serv Res*. 2018;18(1):181–181.

5. Anderson LM, Petticrew M, Rehfuess E, Armstrong R, Ueffing E, Baker P, et al. Using logic models to capture complexity in systematic reviews. *Res Synth Methods* [Internet]. 2011;2(1):33–42. Available from: http://dx.doi.org/10.1002/jrsm.32

6. Menon D, Stafinski T. Health technology assessment in Canada: 20 years strong? *Value Health J Int Soc Pharmacoeconomics Outcomes Res*. 2009 Jun;12 Suppl 2:S14-19.

7. Vreman RA, Mantel-Teeuwisse AK, Hövels AM, Leufkens HGM, Goettsch WG. Differences in Health Technology Assessment Recommendations Among European Jurisdictions: The Role of Practice Variations. Value Health [Internet]. 2020 Jan 1;23(1):10–6. Available from: https://www.sciencedirect.com/science/article/pii/S1098301519323411

8. Espinoza MA, Cabieses B. [Equity in health and health technology assessment in Chile]. *Rev Med Chil.* 2014 Jan;142 Suppl:S45-9.

9. Panteli D, Kreis J, Busse R. CONSIDERING EQUITY IN HEALTH TECHNOLOGY ASSESSMENT: AN EXPLORATORY ANALYSIS OF AGENCY PRACTICES. *Int J Technol Assess Health Care* [Internet]. 2015/12/29 ed. 2015;31(5):314–23. Available from: https://www.cambridge.org/core/product/2AA3E46053202358B9CC83DC35E1042D

10. Facey Helle KM, Hansen P, Single ANV, European Patients’ Forum, Facey Helle KM, Hansen P, et al. Patient Involvement in Health Technology Assessment [Internet]. Springer N. Singapore: Springer Singapore; 2017. 49 p. (Patient Involvement in Health Technology Assessment; vol. 32). Available from: http://link.springer.com/10.1007/978-981-10-4068-9

11. Frank L, Morton SC, Guise JM, Jull J, Concannon TW, Tugwell P, et al. Engaging Patients and Other Non-Researchers in Health Research: Defining Research Engagement. *J Gen Intern Med.* 2020;35(1):307–14.

12. Abelson J, Tripp L, Kandasamy S, Burrows K. Supporting the evaluation of public and patient engagement in health system organizations: Results from an implementation research study. *Health Expect Int J Public Particip Health Care Health Policy.* 2019 Oct;22(5):1132–43.

13. Gagnon MP, Tantchou Dipankui M, Poder TG, Payne-Gagnon J, Mbemba G, Beretta V. Patient and public involvement in health technology assessment: update of a systematic review of international experiences. *Int J Technol Assess Health Care.* 2021 Feb 5;37:e36.

14. Mercer RE, Chambers A, Mai H, McDonald V, McMahon C, Chan KKW. Are We Making a Difference? A Qualitative Study of Patient Engagement at the pan-Canadian Oncology Drug Review: Perspectives of Patient Groups. *Value Health J Int Soc Pharmacoeconomics Outcomes Res*. 2020;23(9):1157–62.

15. Concannon TW, Grant S, Welch V, Petkovic J, Selby J, Crowe S, et al. Practical Guidance for Involving Stakeholders in Health Research. *J Gen Intern Med* [Internet]. 2019 Mar 1;34(3):458–63. Available from: https://doi.org/10.1007/s11606-018-4738-6

16. Adkins EM, Nicholson L, Floyd D, Ratcliffe M, Chevrou-Severac H. Oncology drugs for orphan indications: how are HTA processes evolving for this specific drug category?. *Clin Outcomes Res CEOR* [Internet]. 2017;9:327–42. Available from: http://ovidsp.ovid.com/ovidweb.cgi?T=JS&PAGE=reference&D=prem&NEWS=N&AN=28652787

17. Facey KM. As health technology assessment evolves so must its approach to patient involvement. *J Comp Eff Res.* 2019;8(8):549–54.

18. Haerry D, Landgraf C, Warner K, Hunter A, Klingmann I, May M, et al. EUPATI and Patients in Medicines Research and Development: Guidance for Patient Involvement in Regulatory Processes. Front Med [Internet]. 2018;5:230–230. Available from: http://ovidsp.ovid.com/ovidweb.cgi?T=JS&PAGE=reference&D=prem2&NEWS=N&AN=30175100
