# Supplementary material for: Patient engagement for the development of equity-focused health technology assessment (HTA) recommendations: a case study of two Canadian HTA organizations
Source: Int J Technol Assess Health Care. 2025 Mar 28;41(1):e27. doi: 10.1017/S0266462325000182 (PMC12086597; doi:10.1017/S0266462325000182)
Supplement: Simeon et al. supplementary material [file S0266462325000182sup001.zip › Supplement 2_Additional Details on Methods_Feb.19.docx]

**Supplement 2: Further Details on Methods**

1. **Rationale for Study Design**

The case study approach was chosen to provide a comprehensive understanding of complex phenomena within their real-world context, particularly the patient engagement processes within these two organizations (1). An explanatory case study was particularly suitable for this research as it allowed for the development of theoretical insights into how different patient engagement strategies might influence the incorporation of equity factors in HTA recommendations (2). This design facilitated the identification of patterns and relationships specific to the Canadian context, where varying governance structures and local healthcare needs may shape the importance and impact of patient engagement on health equity. By focusing on the unique contexts of federal and provincial HTA practices, the study aimed to generate meaningful evidence that could inform the development of more equity-sensitive HTA recommendations across different settings (2).

The context-bound nature of HTA, particularly in the Canadian setting, is critical because governance structures, healthcare priorities, and population needs can influence the relevance of patient engagement and HTA practices, including emphasizing health equity considerations in recommendations (3–7). In Canada, healthcare delivery and policy decisions are primarily the responsibility of provincial and territorial governments, leading to variations in how HTA is conducted across jurisdictions (8,9).

Understanding these context-specific nuances is essential because it can highlight how local needs, available resources, and governance priorities shape the HTA process, influencing which patient voices are heard and how equity is addressed. This context-bound approach allows for tailored recommendations that are relevant and actionable within the specific healthcare environment, enhancing the impact of HTA on decision-making and ultimately leading to more equitable health outcomes. By studying these patterns and relationships in context, researchers can identify best practices and challenges unique to HTA practices, providing valuable insights to inform broader applications while acknowledging the diversity of healthcare needs and governance structures (8,10,11).

1. **Sample Size Calculation**

We selected a purposeful sample of 60 HTA reports containing patient input published between 2013 and 2021 from CDA and Ontario Health. The decision to focus on Canadian HTA reports is grounded in the understanding that HTA processes are highly context-bound, with significant variability in patient engagement strategies across organizations and countries (8,10,11). This context specificity means that including reports from non-comparable settings could compromise the accuracy of our findings and limit their generalizability (8,10,11).

The sample size was determined based on the adequacy required for logistic regression analysis, drawing on existing literature and prior studies. A previous study analyzing equity considerations across 19 HTA agencies found that approximately 50% of these agencies incorporated equity factors through their methodologies or ethical analysis (10). Another study on including health equity factors in the World Health Organization (WHO) guidelines revealed that only 25% of the guidelines included PROGRESS-Plus items (12). Anticipating a higher proportion of equity-focused recommendations in Canadian HTA reports due to the requirement to consider local context, we estimated that 40% of the reports would include equity factors, leading to an initial sample size calculation of 50 reports. We increased the sample size to 60 reports to account for variability and ensure robustness. Given its localized focus, this approach reflects our expectation that HTA would address equity factors more comprehensively than WHO guidelines, which often need further adaptation before being implemented nationally.

1. **Identification of Eligible HTA Reports**
   1. **Inclusion Criteria**

HTA reports had to meet specific criteria to be included in this study. First, HTA organizations must involve patients in creating the reports. Second, the reports should have clear recommendations, but they were not required to include health equity factors in their recommendations. Third, eligible HTA reports must have been published between 2013 and 2021.

- 1. **Exclusion Criteria**

Reports were excluded if healthcare providers provided input on behalf of patients, if patient experience reviews were used as a substitute for patient input, or if reports did not include any input. RS identified the HTA organizations and the HTA reports. RS and AA screened all the reports for eligibility.

- 1. **Characteristics of Included Studies**

## **Identification of HTA Organization**

We focused on HTA reports from CDA and Ontario Health for this explanatory case study after searching the International Network of Agencies for Health Technology Assessment (INHATA) database (13). We also scanned the websites of five health ministries in Canada to find publicly available HTA reports that used patient engagement and were published in English. Our search revealed that including recommendations in HTA reports varies with different healthcare governance structures. In British Columbia, Alberta, Manitoba, Ontario, and Newfoundland and Labrador, HTA reports often provide evidence summaries without explicit recommendations. While Canada has universal healthcare coverage, the responsibility for healthcare delivery remains with the provinces and territories (9). These practices reflect variations in HTA processes that may affect patient engagement approaches and the integration of health equity factors into HTA recommendations (3,5,10).

Canada’s Drug Agency (CDA) and Ontario Health consistently publicly published HTA reports with recommendations. Researchers and patients consulted for this study expressed the need to evaluate the impact of patient engagement practices on promoting health equity through HTA. Given their consistent publication of HTA reports with recommendations, CDA and Ontario Health were selected as case studies. Their practices can help draw insights to inform strategies for promoting the integration of equity-focused recommendations for diverse populations across various regions and countries.

- - 1. **Identification of HTA Reports**

We selected reports based on types of HTA reviews, years of publications, and patient engagement. Contrary to Ontario Health, which did not categorize HTA products on its website, CDA had several HTA products. Two CDA products were selected: the Common Drug Review (CDR) and the pan-Canadian Oncology Drug Review (pCODR). The term “common drugs” designates health technologies in the CDA Common Drug Review (CDR), which covers health technologies for conditions such as hypertension, diabetes, and asthma.

We selected the 2013-2021 period to identify HTA reports from CDA before establishing the Patient and Community Liaison Forum, which was created to improve patient involvement in HTA processes (31). Ontario Health began including patient input in its HTA reports in 2015. This timeline can also help capture the evolution of patient engagement in HTA practices.

The flow diagram in Figure 1 outlines the systematic approach used to select HTA reports from Canada's Drug Agency (CDA) and Ontario Health. The process began with identifying three categories of HTA reviews: the Common Drug Review (CDR), the pan-Canadian Oncology Drug Review (pCODR), and reports from Ontario Health focusing on medical devices and virtually delivered health technologies. Using stratified sampling, 60 reports were randomly selected across the three categories: twenty-five from CDR, fifteen from pCODR, and twenty from Ontario Health.


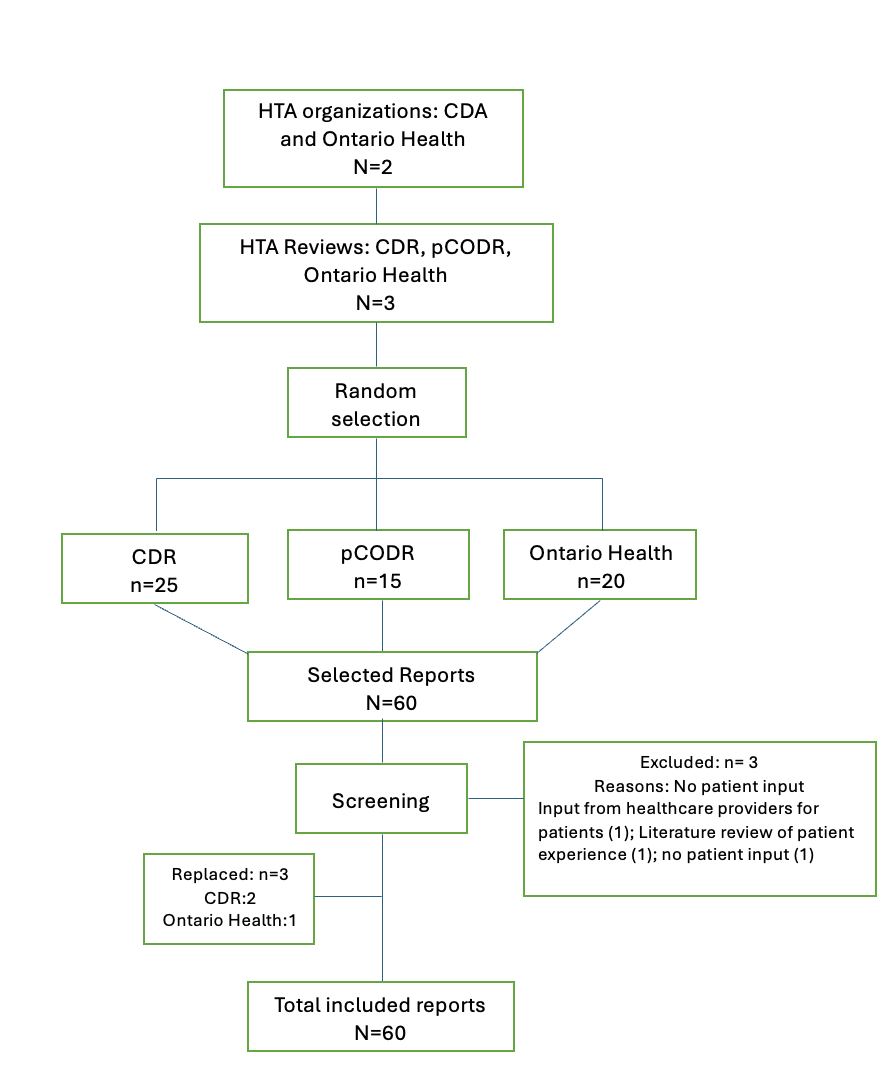


Figure 1: Flow Diagram for the Identification and Inclusion of Studies.

Stratified sampling enhances the sample's representativeness, ensuring that all relevant HTA reports are included. This approach minimizes selection bias, increasing the validity and reliability of the study’s findings by capturing a broad spectrum of recommendations across HTA categories (15). This sampling approach can help identify valuable insights into these organizations' practices for developing equity-focused recommendations. This method will help strengthen the generalizability of the findings, making them applicable to broader HTA practices both within Canada and internationally (15).

1. **Screening**

During the screening phase, reports were carefully reviewed to confirm the presence of patient engagement and HTA recommendations. Three reports were excluded due to the absence of patient engagement—one included feedback from healthcare providers, one was based on a literature review of patient experiences, and one had no patient input. The three reports were replaced to maintain the sample's integrity: two from CDR and one from Ontario Health. Studies were not screened based on the presence of health equity factors in their recommendations. The final sample included 60 HTA reports that met the study’s criteria.

1. **Data extraction**
   1. **Description of variables of interest**

We reviewed the patient input section of the reports and patient engagement guidelines from the CDA and Ontario Health websites to identify key parameters of patient engagement. We identified the types, modes, and modalities of patient engagement and patients' decision-making models and roles in the HTA process. We described patient engagement activities with items from the practical guidance for involving stakeholders in health research (13). We identified equity-focused HTA recommendations based on the PROGRESS-Plus framework (16), the checklist to guide equity considerations in HTA (17) and the published literature on characterizing health equity factors in studies (18,19). A single reviewer (AA) conducted data extraction, and the first author (RS) checked the data for quality control.

- 1. **Characteristics of Patient Engagement Processes**

**Types of Engagement***:* This defines how HTA organizations collect patient input.

- *Direct Engagement*: Involves HTA organizations in collecting patient input from individual patients through interviews, surveys, or focus groups.
- *Indirect Engagement*: Involves HTA organizations in collecting patient input through patient organizations.

**Modes of Engagement:** This refers to the methods used by HTA or patient organizations to collect patient input. These may include interviews, surveys, focus groups, and mixed methods (a combination of surveys, interviews, and focus groups).

**Modalities of Engagement:** Refers to the means used to facilitate engagement, such as digital platforms, telephones, or in-person meetings.

**Decision-Making Models:** Describes the approach HTA organizations used to incorporate patient input into HTA recommendations. The HTA committees used consensus meetings and voting.

**Patient Roles:** Defines the capacity in which patients were involved in the HTA process, either as key informants or as members of HTA advisory committees.

**Timing of engagement:** HTA organizations identify when patients are involved in the HTA process, including scoping, evidence gathering, and decision-making to assess the impact of patient engagement.

- 1. **Characteristics of Equity-Focused HTA Recommendations**

**Equity-Focused HTA Recommendation:** Defined as a recommendation that includes considerations of at least one PROGRESS-Plus factor (Place of residence, Race/ethnicity, Occupation, Gender, Religion, Education, Socioeconomic status, Social capital, plus other relevant stratifying factors such as age, disability, logistics of treatment, severity of conditions and sexual orientation).

- 1. **Characteristics of Included HTA Reports**

**Year of Publication:** Reports were classified into two periods, 2013-2015 (earlier implementation) and 2016-2021 (recent implementation), reflecting different stages of patient engagement evolution in HTA.

**Types of HTA Review:** The reports are categorized into three groups: pan-Canadian Oncology Drug Review (pCODR) for cancer drugs, Common Drug Review (CDR) for non-cancer drugs, and Ontario Health reports on medical devices and virtually delivered health technologies. HTA reports were not categorized into pharmaceuticals and non-pharmaceuticals to focus on patient engagement processes, informing the development of equity-focused HTA recommendations more broadly.

1. **Data Management and Analysis**

The data extraction process for this study followed a systematic approach to ensure accurate and comprehensive capture of relevant data from the chosen HTA reports. To start, we created a detailed data extraction form in Excel, designed to capture critical variables such as types, modes, and modalities of patient engagement, decision-making models, and the roles of patients in the HTA process. This form was aligned with the PROGRESS-Plus framework, a checklist on health equity in HTA, and established literature on patient engagement practices. All selected HTA reports were rigorously reviewed during the screening phase to confirm eligibility. Reports that did not meet the inclusion criteria, such as those lacking direct patient input or including only healthcare provider perspectives, were excluded from further analysis.

Data extraction was carried out in two stages to ensure accuracy and reliability. First, one reviewer extracted data from each HTA report, recording information on patient engagement processes, decision-making models, and whether the recommendations included equity considerations based on the PROGRESS-Plus factors. This was followed by a quality control step, where a second reviewer cross-checked the extracted data for consistency and accuracy. Any discrepancies among the reviewers were resolved through discussion, with input from senior authors, if needed, to reach an agreement. This data extraction approach helped ensure the validity and reliability of the findings.

Data management and analysis were conducted using Excel for descriptive statistics and R software for inferential analysis. Pearson’s chi-squared tests assessed the associations between patient engagement variables and equity-focused HTA recommendations. In contrast, logistic regression was used to examine the direction and strength of these relationships.

Descriptive statistics were applied to summarize the key characteristics of patient engagement processes, encompassing the types, methods, and modalities of patient engagement, decision-making models, and patient roles. This approach facilitated a foundational understanding of the documented engagement practices in the HTA reports.

**References:**

1. Carey TS, Sanders GD, Viswanathan M, Trikalinos TA, Kato E, Chang S. Taxonomy for Study Designs. 2012; Available from: https://www.ncbi.nlm.nih.gov/books/NBK95280/

2. Fisher I, Ziviani J. Explanatory case studies: Implications and applications for clinical research. Aust Occup Ther J [Internet]. 2004 Dec 1 [cited 2023 Sep 1];51(4):185–91. Available from: https://doi.org/10.1111/j.1440-1630.2004.00446.x

3. Espinoza MA, Cabieses B. [Equity in health and health technology assessment in Chile]. Rev Med Chil. 2014 Jan;142 Suppl:S45-9.

4. Facey KM, Bedlington N, Berglas S, Bertelsen N, Single ANV, Thomas V. Putting Patients at the Centre of Healthcare: Progress and Challenges for Health Technology Assessments. The patient [Internet]. 2018;11(6):581–9. Available from: http://ovidsp.ovid.com/ovidweb.cgi?T=JS&PAGE=reference&D=prem&NEWS=N&AN=30051315

5. Gagnon MP, Tantchou Dipankui M, Poder TG, Payne-Gagnon J, Mbemba G, Beretta V. Patient and public involvement in health technology assessment: update of a systematic review of international experiences. Int J Technol Assess Health Care. 2021 Feb 5;37:e36.

6. Snow ME, Tweedie K, Pederson A. Heard and valued: the development of a model to meaningfully engage marginalized populations in health services planning. BMC Health Serv Res. 2018;18(1):181–181.

7. Vreman RA, Mantel-Teeuwisse AK, Hövels AM, Leufkens HGM, Goettsch WG. Differences in Health Technology Assessment Recommendations Among European Jurisdictions: The Role of Practice Variations. Value Health [Internet]. 2020 Jan 1;23(1):10–6. Available from: https://www.sciencedirect.com/science/article/pii/S1098301519323411

8. Menon D, Stafinski T. Health technology assessment in Canada: 20 years strong? Value Health J Int Soc Pharmacoeconomics Outcomes Res. 2009 Jun;12 Suppl 2:S14-19.

9. Martin D, Miller AP, Quesnel-Vallée A, Caron NR, Vissandjée B, Marchildon GP. Canada’s universal health-care system: achieving its potential. The Lancet [Internet]. 2018 Apr 28 [cited 2022 Nov 25];391(10131):1718–35. Available from: https://doi.org/10.1016/S0140-6736(18)30181-8

10. Busse R, Panteli D, Kreis J, Busse R. Considering equity in health technology assessment: an exploratory analysis of agency practices. Int J Technol Assess Health Care. 2015 Jan;31(5):314–23.

11. Abelson J, Tripp L, Kandasamy S, Burrows K. Supporting the evaluation of public and patient engagement in health system organizations: Results from an implementation research study. Health Expect Int J Public Particip Health Care Health Policy. 2019 Oct;22(5):1132–43.

12. Dewidar O, Tsang P, León-García M, Mathew C, Antequera A, Baldeh T, et al. Over half of the WHO guidelines published from 2014 to 2019 explicitly considered health equity issues: a cross-sectional survey. J Clin Epidemiol. 2020 Nov;127:125–33.

13. Concannon TW, Grant S, Welch V, Petkovic J, Selby J, Crowe S, et al. Practical Guidance for Involving Stakeholders in Health Research. J Gen Intern Med [Internet]. 2019 Mar 1;34(3):458–63. Available from: https://doi.org/10.1007/s11606-018-4738-6

14. MacPhail E, Shea, B. An Inside Look at the Early History of the CADTH Common Drug Review in Canada [Internet]. Canadian Agency for Drugs and Technologies in Health; 2017 Apr [cited 2022 Jun 1] p. 68. Available from: https://www.cadth.ca/sites/default/files/pdf/early_history_of_CDR.pdf

15. Parsons VL. Stratified Sampling. In: Wiley StatsRef: Statistics Reference Online [Internet]. 2017 [cited 2024 Sep 10]. p. 1–11. Available from: https://doi.org/10.1002/9781118445112.stat05999.pub2

16. O’Neill J, Tabish H, Welch V, Petticrew M, Pottie K, Clarke M, et al. Applying an equity lens to interventions: Using PROGRESS ensures consideration of socially stratifying factors to illuminate inequities in health. J Clin Epidemiol [Internet]. 2014;67(1):56–64. Available from: http://dx.doi.org/10.1016/j.jclinepi.2013.08.005

17. Benkhalti M, Espinoza M, Cookson R, Welch V, Tugwell P, Dagenais P. Development of a checklist to guide equity considerations in health technology assessment. Int J Technol Assess Health Care. 2021;37(gti, 8508113):e17.

18. Flanagin A, Frey T, Christiansen SL, AMA Manual of Style Committee. Updated Guidance on the Reporting of Race and Ethnicity in Medical and Science Journals. JAMA [Internet]. 2021 Aug 17 [cited 2022 Nov 20];326(7):621–7. Available from: https://doi.org/10.1001/jama.2021.13304

19. Hosking J, Macmillan A, Jones R, Ameratunga S, Woodward A. Searching for health equity: validation of a search filter for ethnic and socioeconomic inequalities in transport. Syst Rev [Internet]. 2019 Apr 11;8(1):94. Available from: https://doi.org/10.1186/s13643-019-1009-5
