# Supplementary material for: Patient engagement for the development of equity-focused health technology assessment (HTA) recommendations: a case study of two Canadian HTA organizations
Source: Int J Technol Assess Health Care. 2025 Mar 28;41(1):e27. doi: 10.1017/S0266462325000182 (PMC12086597; doi:10.1017/S0266462325000182)
Supplement: Simeon et al. supplementary material [file S0266462325000182sup001.zip › Supplement 3_ Additional Results_Feb.19.docx]

**Supplement 3: Additional Results**

**1. Overview of Patient Engagement Processes**

The analysis of sixty HTA reports from Canada’s Drug Agency (CDA) and Ontario Health revealed diverse patient engagement processes, highlighting direct and indirect methods. The direct engagement involved collecting patient input directly from individual patients, which was predominantly utilized by Ontario Health (thirty-three percent; twenty out of sixty reports). Indirect engagement, primarily used by CDA, involved receiving patient input through submissions from patient organizations, which accounted for sixty-seven percent (forty out of sixty reports) of the total included reports. A minority of reports (twelve percent; seven out of sixty) also included healthcare providers and patient input.

**Table 1: Summary of Patient Engagement Processes in Ontario Health, pCODR and CDR.**

| **Types of HTA review** | **Characteristics** | **Description** | **n (%)** |
| --- | --- | --- | --- |
| **Types of patient engagement** | |  |  |
| Ontario Health | Direct engagement | HTA organizations directly interviewed individual patients to collect patient input | 20 (33%) |
| CDR and pCODR | Indirect engagement | HTA organizations collected patient input through a submission from patient organizations | 40 (67%) |
| **Modes of engagement** | |  |  |
| Ontario Health | Interview | HTA analysts conducted interviews with individual patients (20 out of 20) | 20 (100%) |
| CDR and pCODR | Survey | Patient organizations used a questionnaire to collect patient input (9 out of 22) | 9 (41%) |
| CDR and pCODR | Mixed methods | Patient organizations used a combination of 2 or 3 of the following to collect patient input: survey, interview, and focus groups (13 out 22) | 13 (55%) |
| CDR and pCODR | Unknown | Patient organizations did not report methods of collecting patient input (18 out of 40) | 18 (45%) |
| **Modalities of engagement** | |  |  |
| Ontario Health and CDA | HTA organizations | HTA organizations use digital technologies for patient engagement activities | 60 (100%) |
| CDR and pCODR | Patient organizations | Number of patient organizations who reported the use of digital technologies (13 out 40) | 13 (31%) |
| CDR and pCODR | Unknown | Number of patient engagement who did not report modalities of engagement (21 out 40) | 21 (69%) |
| **Models of decision-making** | |  |  |
| Ontario Health, and pCODR | Consensus | All members of the HTA advisory committee can contribute to the decision | 35 (58%) |
| CDR | Vote | All members of the HTA advisory committee can vote on the decision | 25 (42%) |
| **Roles of patient** | |  |  |
| Ontario Health, CDR and pCODR | Key informants | The patient provided their feedback to HTA or patient organizations | 60 (100%) |
|  | Members of an advisory committee | The patient contributed to the decision |  |
| **Timing of engagement** | | | |
| Ontario Health, CDR and pCODR | During effectiveness analysis and decision-making | At what stage of the HTA process patient input is elicited | 60 (100%) |

1. **Modes and Modalities of Engagement**

The modes of engagement varied between interviews, surveys, and mixed methods. Interviews were the primary mode for Ontario Health, and they were used in all its reports (one hundred percent; twenty out of twenty). In contrast, among the patient organizations submitting input to CDA, fifty-five percent (twenty-two out of forty) reported their methods of collecting feedback. Of these, sixty percent (thirteen out of twenty-two) utilized mixed methods (surveys, interviews, focus groups), while forty percent (nine out of twenty-two) relied solely on surveys. Digital technologies were the primary modality for engaging patients. Ontario Health and CDA employed digital tools such as online surveys, discussion boards, and social media to facilitate engagement.

1. **Decision-Making Models and Patients’ Roles**

The decision-making models identified in the reports included consensus meetings and voting. Consensus was the predominant decision-making model used in fifty-eight percent (thirty-five out of sixty reports) of the HTA processes, particularly within Ontario Health and the pan-Canadian Oncology Drug Review. Voting was utilized primarily in the Common Drug Review processes, accounting for forty-two percent (twenty-five out of sixty reports). Patients contributed as key informants or members of advisory committees and participated in decision-making sessions.

1. **Characteristics of equity-focused HTA recommendations**

We identified twelve unique PROGRESS-Plus items across all the included HTA reports, six of which were from the PROGRESS category. These consisted of a place of residence, language, gender, education, socioeconomic status, and social capital. We coded the other six items in the “Plus” category. They consisted of affordability, age, ethical issues, the conditions' severity, treatment logistics, and stigma. We recorded stigma, social capital, and gender in patient input only. We did not find the following items from the PROGRESS framework—race, ethnicity, culture, and religion—in any sections of the included HTA reports.

As shown in Table 2, Ontario Health had the highest proportion of HTA reports with equity-focused HTA recommendations (seventy-five percent; fifteen out of twenty reports). The pan-Canadian Oncology Drug Review (sixty-seven percent; ten out of fifteen reports) and the Common Drug Review (sixty-four percent; sixteen out of twenty-five reports) came in second and third position, respectively.

**Table 2: Equity-focused HTA recommendations in the included reports.**

| Types of HTA review | Types of patient engagement | Models of decision-making | N=60 | Equity-focused HTA recommendations n (%) |
| --- | --- | --- | --- | --- |
| Ontario Health | Direct | Consensus | 20 | 15 (75%) |
| pan-Canadian Oncology Drug Review (pCODR) | Indirect | Consensus | 15 | 10 (67%) |
| Common Drug Review (CDR) | Indirect | Voting | 25 | 16 (64%) |

1. **Health equity factors in patient input and HTA recommendations**

We compared the number of PROGRESS-Plus items identified in patient input with those recorded in HTA recommendations. As shown in Figure 1, mentions of PROGRESS-Plus items were more common in the patient input section (total mentions: eighty-four; range one to seven) than in the HTA recommendation section of the reports (full mentions: seventy-two; range one to four).

**Figure 1: PROGRESS-Plus Mentions in the Included Reports.**


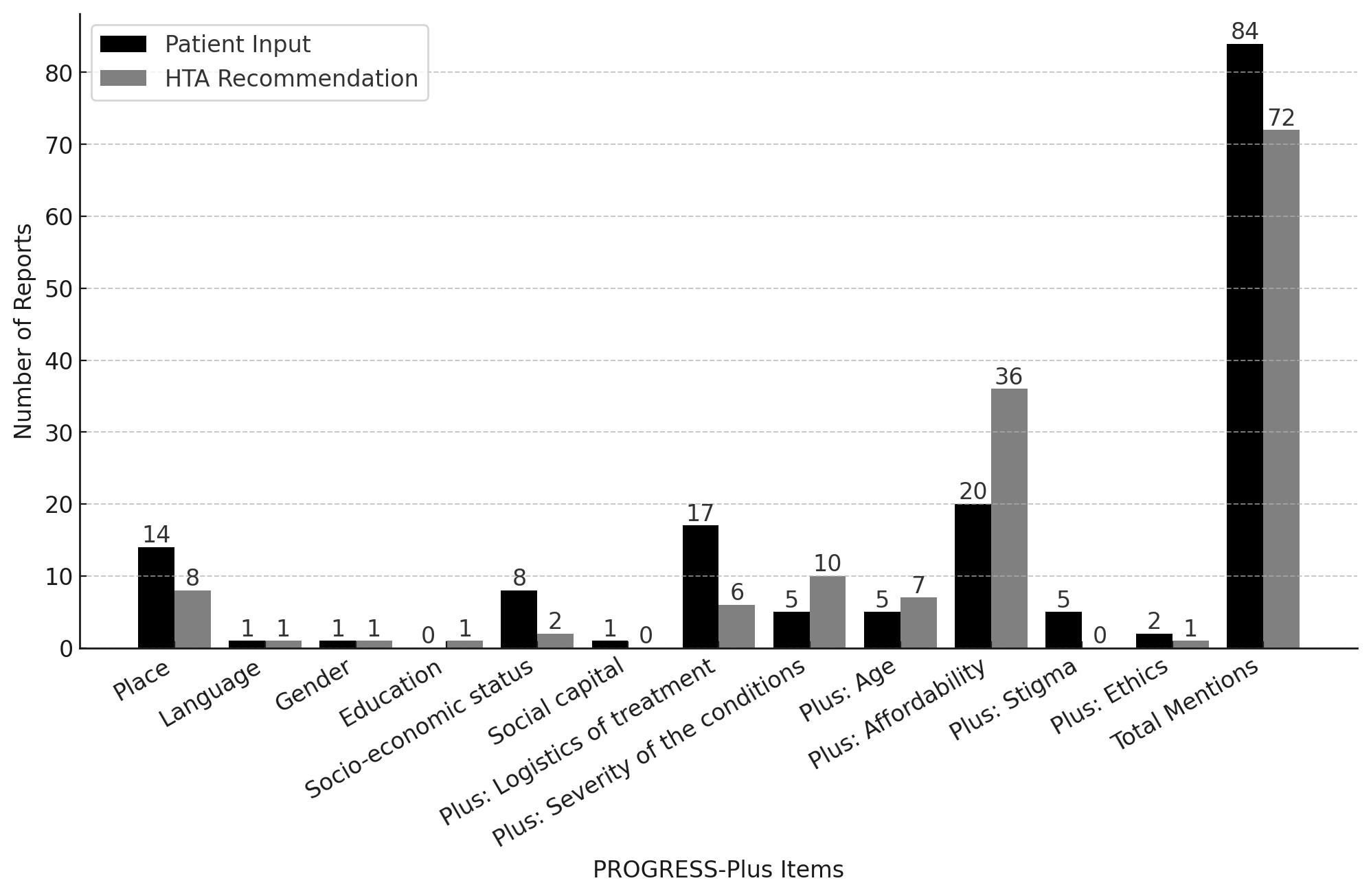


We identified a total of eight PROGRESS-Plus items that are common to both patient input and HTA recommendation sections of the reports. The frequency of the top six of these PROGRESS-Plus items is distributed as follows in patient input and HTA recommendations, respectively:

1. Affordability: sixty percent (twenty out of thirty-three reports) compared to eighty-seven percent (thirty-six out of forty-one reports).
2. Logistics of treatment: fifty-one percent (seventeen out of thirty-three reports) compared to fifteen percent (six out of forty-one reports).
3. Place of residence: forty-two percent (fourteen out of thirty-three reports) compared to nineteen percent (eight out of forty-one reports).
4. Socioeconomic status: twenty-four percent (eight out of thirty-three reports) compared to zero point five percent (two out of forty-one reports).
5. Severity of conditions: fifteen percent (five out of thirty-three reports) compared to twenty-four percent (ten out of forty-one reports).
6. Age: fifteen percent (five out of thirty-three reports) compared to seventeen percent (seven out of forty-one reports).
